# Supplementary material for: Phylogeny and Systematics of the Genus Tolypocladium (Ophiocordycipitaceae, Hypocreales)
Source: J Fungi (Basel). 2022 Nov 1;8(11):1158. doi: 10.3390/jof8111158 (PMC9697939; doi:10.3390/jof8111158)
Supplement: Supplementary file 1 [file jof-08-01158-s001.zip › Supplementary Figure S1.pdf]

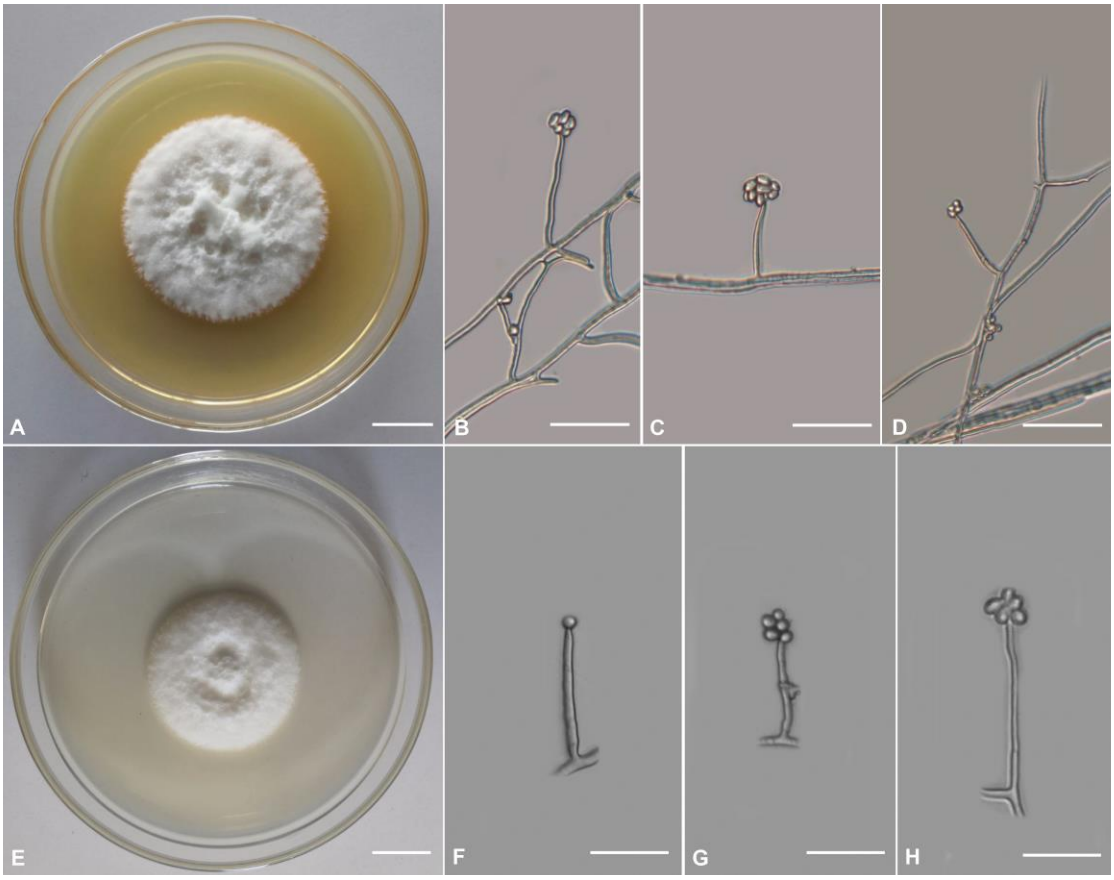

Figure S1. Morphology of *Tolypocladium subparadoxum* NBRC 106958 *and Tolypocladium paradoxum* NBRC 100945 (**A–D**: *T. subparadoxum* NBRC 106958. **E–H**: *T. paradoxum* NBRC 100945) (**A**) Culture character on PDA medium incubated at 22°C for 21 days; (**B–D**) Phialides and conidia. (**E**) Culture character on PDA medium incubated at 22°C for 14 days; (**F–H**) Phialides and conidia. Scale bars: A, E = 10 mm; B–D = 30 μm; F–H = 20 μm.
