# Supplementary material for: Phylogeny and Systematics of the Genus Tolypocladium (Ophiocordycipitaceae, Hypocreales)
Source: J Fungi (Basel). 2022 Nov 1;8(11):1158. doi: 10.3390/jof8111158 (PMC9697939; doi:10.3390/jof8111158)
Supplement: Supplementary file 1 [file jof-08-01158-s001.zip › Supplementary Tables.pdf]

Table S1. Pairwise genetic distance matrix of *Tolypocladium* species for *tef-1α* sequences.

Taxon	1	2	3	4	5	6	7	8	9	10
1 (*T. capitatum* YFCC 881)										
2 (*T. cylindrosporum* ARSEF 2920)	0.055									
3 (*T. inflatum* OSC 71235)	0.059	0.030								
4 (*T. japonicum* NBRC 9647)	0.050	0.048	0.061							
5 (*T. jezoense* NBRC 106328)	0.042	0.040	0.041	0.032						
6 (*T. ophioglossoides* NBRC 100998)	0.043	0.041	0.047	0.044	0.032					
7 (*T. paradoxum* YFCC 882)	0.048	0.034	0.037	0.042	0.028	0.032				
8 (*T. pseudoalbum* YFCC 875)	0.066	0.076	0.079	0.078	0.075	0.072	0.070			
9 (*T. reniformisporum* YFCC 1805002)	0.056	0.026	0.039	0.050	0.046	0.047	0.041	0.078		
10 (*T. subparadoxum* YFCC 879)	0.044	0.043	0.050	0.045	0.030	0.033	0.027	0.072	0.045	

Table S2 . Pairwise genetic distance matrix of *Tolypocladium* species for partial *ITS* sequences.

Taxon	1	2	3	4	5	6	7	8	9	10
1 (*T. capitatum* YFCC 881)										
2 (*T. cylindrosporum* ARSEF 2920)	0.055									
3 (*T. inflatum* OSC 71235)	0.050	0.008								
4 (*T. japonicum* NBRC 9647)	0.087	0.074	0.075							
5 (*T. jezoense* NBRC 106328)	0.064	0.057	0.057	0.079						
6 (*T. ophioglossoides* NBRC 100998)	0.045	0.029	0.025	0.077	0.055					
7 (*T. paradoxum* YFCC 882)	0.064	0.045	0.045	0.084	0.059	0.047				
8 (*T. pseudoalbum* YFCC 875)	0.072	0.064	0.060	0.095	0.072	0.055	0.052			
9 (*T. reniformisporum* YFCC 1805002)	0.053	0.014	0.014	0.064	0.055	0.027	0.043	0.057		
10 (*T. subparadoxum* YFCC 879)	0.053	0.021	0.021	0.075	0.038	0.034	0.036	0.050	0.019	

Table S3. Pairwise genetic distance matrix of *Tolypocladium* species for partial *rpb1* sequences.

Taxon	1	2	3	4	5	6	7	8	9	10
1 (*T. capitatum* YFCC 881)										
2 (*T. cylindrosporum* ARSEF 2920)	0.086									
3 (*T. inflatum* OSC 71235)	0.065	0.032								
4 (*T. japonicum* NBRC 9647)	0.070	0.067	0.047							
5 (*T. jezoense* NBRC 106328)	0.077	0.076	0.058	0.051						
6 (*T. ophioglossoides* NBRC 100998)	0.068	0.074	0.061	0.060	0.065					
7 (*T. paradoxum* YFCC 882)	0.071	0.065	0.052	0.051	0.065	0.056				
8 (*T. pseudoalbum* YFCC 875)	0.133	0.119	0.103	0.118	0.117	0.135	0.118			
9 (*T. reniformisporum* YFCC 1805002)	0.094	0.017	0.038	0.074	0.083	0.080	0.074	0.123		
10 (*T. subparadoxum* YFCC 879)	0.092	0.080	0.071	0.071	0.092	0.086	0.046	0.148	0.089	

Table S4. Pairwise genetic distance matrix of *Tolypocladium* species for partial *rpb2* sequences.

Taxon	1	2	3	4	5	6	7	8	9	10
1 (*T. capitatum* YFCC 881)										
2 (*T. cylindrosporum* ARSEF 2920)	0.072									
3 (*T. inflatum* OSC 71235)	0.070	0.037								
4 (*T. japonicum* NBRC 9647)	0.071	0.063	0.063							
5 (*T. jezoense* NBRC 106328)	0.062	0.065	0.058	0.034						
6 (*T. ophioglossoides* NBRC 100998)	0.056	0.055	0.053	0.045	0.048					
7 (*T. paradoxum* YFCC 882)	0.077	0.060	0.057	0.052	0.052	0.047				
8 (*T. pseudoalbum* YFCC 875)	0.134	0.101	0.109	0.108	0.112	0.104	0.110			
9 (*T. reniformisporum* YFCC 1805002)	0.076	0.013	0.042	0.066	0.068	0.060	0.062	0.100		
10 (*T. subparadoxum* YFCC 879)	0.075	0.053	0.053	0.051	0.054	0.047	0.024	0.108	0.052	
